# Liston’s Elements of Surgery—New Edition

**Published:** 1842-05

**Authors:** 


### LISTON'S ELEMENTS OF SURGERY—NEW EDITION.

Messrs. Barrington & Haswell, Philadelphia, have in press a new edition of that deservedly popular book, Liston's Elements of Surgery, with notes and additions by Dr. Gross, the accomplished Professor of Surgery in the Louisville Medical Institute. The work will be illustrated by numerous wood-cuts, and be comprised in an octavo volume of five or six hundred pages. It will appear early in June, when we shall take occasion to speak of it more fully. C.

